# A balancing act: integrating the expertise of youth peer workers in child and adolescent mental health services

**DOI:** 10.1007/s00787-024-02498-4

**Published:** 2024-06-15

**Authors:** C. R. M. de Beer, R. R. J. M. Vermeiren, L. A. Nooteboom, C. H. Z. Kuiper, J. C. M. L. Groenendijk, M. de Vreugd, L. van Domburgh

**Affiliations:** 1 LUMC Curium-Department of Child and Adolescent Psychiatry, Leiden University Medical Center, Post Box 15, 2300 AA Leiden, The Netherlands; 2 Amsterdam University Medical Center (AUMC), Amsterdam, the Netherlands; 3 iHUB, Alliance of Youth Care, Mental Health Care, and Educational Organizations, Rotterdam, The Netherlands; 4 Department of Child Development and Education, University of Amsterdam, Amsterdam, the Netherlands

**Keywords:** Lived experience workforce, Youth peer support workers, Child and adolescent mental health services, Youth

## Abstract

The socio-relational focus of youth peer support workers (YPSWs) poses a challenge when YPSWs are embedded in medical oriented contexts common to child and adolescent mental health services (CAMHS); as it requires YPSWs to find a balance between being a peer on one hand, and adhering to professional boundaries and medical standards set out by CAMHS on the other. To create a suitable position for YPSWs in CAMHS, this study investigated the unique socio-relational contributions YPSWs can make to CAMHS in addition to clinicians, and identified how these contributions can be embedded within CAMHS. This study reports on 37 semi-structured interviews conducted in the Netherlands with youth (n = 10), YPSWs (n = 10), and clinicians (n = 17). Overall, the unique socio-relational contributions YPSWs can make include: their ability to build authentic trusting relationships with youth by providing empowerment, promoting autonomy, valuing stillness in recovery, reducing isolation, recognizing strengths, and navigating life inside and outside of (residential) mental healthcare and beyond classification. Moreover, prerequisites to safeguard the integration of YPSWs and these socio-relational contributions were also identified, including YPSWs achieving stability in recovery, recent lived experiences with mental health challenges, and organizational support in terms of suitable treatment climate, resources to enhance flexibility of YPSWs, and shared goals regarding youth peer support work. Overall, YPSWs view youth holistically and foster a connection with youth based on youthfulness and recent lived experience. Involving YPSWs is an important step forward to drive positive transformation in CAMHS.

**Supplementary Information:**

The online version contains supplementary material available at 10.1007/s00787-024-02498-4.

## Introduction

The integration of youth peer support workers (YPSWs) in child and adolescent mental health services (CAMHS) is expanding and becoming more widely accepted [1].YPSWs are young adults with lived experience of mental health challenges who provide social, emotional and practical support to youth with mental health challenges [2]. YPSWs can place value on equality and can support youth to find personal meaning during recovery from mental health challenges [1–3]. In doing so, YPSWs may add substantially to the care of youth who experience mental health challenges [1, 3]. This is important as youth with mental health challenges often feel existing CAMHS do not fully meet their needs, and services are too fixated on traditional clinical models to treat psychiatric symptomatology [2, 4–6]. Involving YPSWs can therefore assist CAMHS in providing mental healthcare that includes developmentally appropriate, youth-centered and recovery-oriented support [7, 8].

While support by YPSWs can be beneficial for youth with mental health challenges, the process of embedding YPSWs in CAMHS poses a significant challenge [2]. Central to this challenge is the socio-relational focus of youth peer support. This socio-relational focus is grounded on sharing personal experiences, mutual understanding, and forming authentic relationships with youth [9, 30]. This enables YPSWs to connect on a personal level with youth and to understand their experiences in ways that some medically oriented mental health workers may not [10, 11]. This socio-relational focus in particular poses a challenge when YPSWs are integrated in medical oriented contexts common to CAMHS; as it requires YPSWs to find a balance between being a peer on one hand, and adhering to professional boundaries and medical standards set out by CAMHS on the other [1, 2, 9]. Moreover, the role of YPSWs as non-clinical peer-based workers may cause confusion by receivers of mental health care, clinicians, and YPSWs themselves, as it may not fit neatly in the medically oriented context common in CAMHS [1, 5, 9]. Thus, to create a suitable position for YPSWs in CAMHS, research is needed to capture the unique socio-relational contributions YPSW can make in addition to clinicians, and to identify how these contributions can be embedded within CAMHS [12]. Research into the components underpinning the socio-relational contributions of YPSWs can enhance our understanding of what YPSWs can bring to CAMHS, and can inform the development of more viable treatment approaches to meet the diverse needs of youth with mental health challenges.

Therefore, the aim of this study is two-fold. Through semi-structured interviews with youth receiving mental health services, clinicians, and YPSWs we aim to: (1) investigate the unique socio-relational contributions YPSWs can make in addition to clinicians in CAMHS; and (2) gain insight in the necessary requirements to safeguard the socio-relational focus of youth peer support in CAMHS.

## Methods

### Study design and setting

This qualitative study presents the outcomes of semi-structured interviews with youth, YPSWs, and clinicians. We conducted qualitative research because it enabled us to study the experiences, contexts and different perspectives of the participants, allowing for an in-depth exploration of the socio-relational contributions YPSWs can make to CAMHS [13, 14]. The Leiden University Medical Center’s Medical Ethics Review Board examined the study and determined that this study is not subject to the Medical Research Involving Human Subjects Act (non-WMO approval number: N21.092). The Medical Ethics Review Board also confirmed that the study adheres to the Dutch Code of Conduct for Research Integrity. This study is conducted in accordance with the consolidated criteria for reporting quality research guidelines (COREQ) [15].

In this study, we focus on child and adolescent mental health services (CAMHS): services that offer inpatient, outpatient, and community care for youth aged 12–21 with (severe) psychological and behavioral challenges. Three government funded CAMHS locations in the Netherlands were involved in this study: Leiden University Medical Center Department of Child and Adolescent Psychiatry (LUMC Curium), iHUB, and Pluryn. LUMC Curium focuses on child and adolescent psychiatric treatment, whereas iHUB and Pluryn provide youth care (care for youth and families in the context of child welfare, foster care and residential treatment programs), special needs education, and support (including housing support) for youth with complex behavioral and psychological needs. For all three locations, we focused on outpatient, community and residential care facilities; facilities providing psychiatric, psychosocial and pharmacological treatment to youth with behavioral and psychological problems. Youth in treatment at LUMC Curium, iHUB or Pluryn commonly present with (comorbid): mood and anxiety disorders, eating disorders, autism spectrum disorder, childhood disruptive behavior disorders, attachment issues, substance abuse disorders, and self-harm problems. Youth can access the above-mentioned services through referral from a general practitioner, school doctor, community care teams, or through other CAMHS.

### Participants

We purposively included three types of participants in our research project: youth with experience of receiving CAMHS, YPSWs, and clinicians. Each group of participants represents an important perspective in CAMHS. Our aim was to include at least 10 participants in each group, and we included more participants, to a maximum of 20 participants in each group, to reach data saturation. The reason for including a minimum of 10 participants and a maximum of 20 participants was to ensure the data analysis process remained manageable and feasible [29]. In our study “saturation” was defined as the stage at which the collected dataset has been thoroughly examined and understood in accordance with the research aims to achieve the intended research outcomes [29]. The combination of these differing perspectives allowed for a rich understanding of the socio-relational contributions of YPSWs, and the necessary requirements needed to embed the unique YPSW role in CAMHS-settings.

#### Youth with experience of receiving CAMHS

We approached youth aged 12–24 with experience of receiving care in CAMHS during adolescence (12–21). We took a convenience sampling approach, the recruitment took place by asking YPSWs and youth we knew to spread the recruitment information via WhatsApp in suitable mental health related WhatsApp groups. Moreover, we also spread the recruitment information at LUMC Curium, iHUB, and Pluryn via personal contacts within these centers. Interested participants directly contacted the first author via the provided email address in the recruitment letter.

#### Youth peer workers

For the purpose of participation in this study, we approached YPSWs with personal experience with mental health challenges and treatment during childhood or adolescence. Additionally, to ensure that the YPSWs were capable of sharing relevant work-related experiences, they had to utilize their lived experience as advocates, advisors, educators, coaches or mentors to make an impact on child and adolescent mental health policy; educate and inform others about mental illness; and/ or to provide support to youth in treatment. YPSW recruitment took place at two locations: The National Youth Council (NYC) and Experienced Experts (ExpEx) in the Netherlands. The NYC consists of a panel of youth who draw upon their personal experiences with mental health challenges to enhance care and societal support for their peers facing similar challenges. ExpEx, on the other hand, is an organization that trains and assigns YPSWs to support youth with mental health challenges. ExpEx also assigns YPSWs to educate (future) clinicians, advise policymakers and mental health services. Both ExpEx and the NYC are independent (volunteer) employment organizations. However, YPSWs employed by ExpEx and/or the NYC often collaborate and undertake assignments at iHUB, Pluryn and LUMC Curium to provide peer support services.

To recruit potential participants linked to these organizations, we utilized a convenience sampling approach by sharing the recruitment message through our personal contacts and asking them to pass it along to individuals they knew. Interested participants either requested their contact person to share their details with us, or directly contacted the first author via the provided email address in the recruitment letter.

#### Clinician

For inclusion in this study, clinicians were required to hold positions in CAMHS as psychiatrists, doctors, psychologists, family therapists, sociotherapists, social workers, program managers, or case managers. Moreover, they had to work with or for youth aged 12–21 years. The recruitment process involved a convenience sampling approach at LUMC Curium, iHUB and Pluryn. Team managers and personal contacts within these services were approached by the first author and asked to assist by spreading the study recruitment letter to potential participants within their organizations. Potential participants either asked their contact person to share their contact details with us, or directly contacted the first author via the provided email address in the recruitment letter.

### Data collection and procedure

#### Interview topics

To formulate topics that needed to be addressed during the interviews on youth peer support, themes for the topic lists with open-ended questions for interviews with youth, YPSWs, and clinicians, were created after conducting two focus groups with YPSWs from the NYC (please see Appendix A for these topic lists). Two of the YPSWs engaged in the focus groups also took part in an interview. During the focus groups, themes related to barriers and facilitators in the integration process of youth peer support services in CAMHS were discussed to identify research gaps. The results of these focus groups were discussed by authors CB, LN, RV, and LD during reflexive meetings to create a final topic list with open-ended questions. Authors LN, RV, and LD possessed relevant clinical experience in CAMHS, enabling them to contribute a clinical perspective during the formulation of these topic lists. A large range of topics were covered during the interviews, including but not limited to: added value of YPSWs in CAMHS, self-disclosure of YPSWs versus clinicians; (organizational) requirements for YPSWs; guidance and support needs of clinicians and YPSWs; and facilitators and barriers in the implementation process and pursuit of youth peer support work. All interviews were conducted in Dutch. Thus, the quotes in the ‘Results’ section may be susceptible to translator bias.

#### Information and consent

The first author e-mailed participants who had expressed interest in participating in an interview with an information letter. After initial contact through e-mail, the first author also e-mailed participants to plan a date for the interview, and requested participants to sign a (digital) informed consent form prior to the interview. We requested participants to provide consent for the storage, recording, transcription, and utilization of pseudonymized data in journal articles and presentations. No participants withdrew their consent during or after application of the interview.

#### Interview procedure

The recruitment for the interviews took place from January 2022 to July 2022. For most participants, except for one young person who preferred to have the interview face-to-face, the interviews were held online through Microsoft Teams between February 2022 and August 2022 due to the COVID restrictions. The interviews lasted between 27 to 66 min. No stipends were provided to the participants in this study. The interviews with youth and YPSWs were carried out by the first author of this study (CB) in collaboration with one of the two YPSWs who also served as co-researchers in this study and listed as co-authors on this paper (fifth and sixth authors of this study—JG and MV). During the interviews with clinicians no co-researchers were present to create an environment where clinicians felt comfortable expressing any concerns they might have had regarding youth peer support. We included a YPSW as a co-researcher, since their sensitivity and non-verbal understanding of youth in CAMHS can put participants at ease, allowing for improved rapport [16]. However, it should be noted that the involvement of YPSWs as co-researcher could also have implications for the results. For example, youth may not have been fully open about negative experiences they had with regards to youth peer support. The co-researchers present during the interviews had prior experience of utilizing their lived experience as YPSWs in the role of advisors, educators and mentors for youth with mental health challenges. All interviews were recorded and transcribed verbatim, and field notes were taken by a student assistant from the Leiden University Medical Center during the interviews. Field notes were mainly taken in case the recording would fail or to capture non-verbal cues that could not be captured by the audio recordings. For most participants, except for one YPSW and three clinicians, the interview was their first interaction with the main interviewer (first author). A total of four youth had previously met with one of the co-researchers (fourth and fifth author). However, none of the youth had previously received support or mentoring from one of the co-researchers present during the interviews. Despite the age range of 12–24, no youth below the age of 16 expressed interest to participate. This could be due to these youth being required to ask for additional consent from their parent or guardian to participate.

#### Main researcher

The main interviewer and researcher is the first author (CB) of this paper. She identifies as female, and was 27–28 years old at the time of the interviews. The researcher has a Master’s degree in psychology and was working as a PhD candidate at LUMC Curium. The researcher has prior experience with research on youth peer support as she had previously conducted a systematic review on youth peer support [2]. She also has prior experience of working with peer support workers during an internship at a center for adults in treatment for addiction. Overall, the researcher is positive towards integrating YPSWs in child and adolescent psychiatry. She sees the added value of YPSWs, but understand there are numerous barriers to overcome during the integration process of YPSWs in CAMHS.

### Analysis

We used Atlas.ti (version 9), a qualitative data software program, for coding and organizing textual data in our analysis of transcribed interviews. Our approach involved reflexive thematic analysis, aligning with the exploratory nature of the study, and enabling thorough engagement, interpretation, analysis, and description of the interview data while considering our own subjectivity. This reflexivity was demonstrated through strategic, relational, and contextual-discursive reflectivity [14]. Strategic reflexivity guided the interviews, analysis and results presentation; we were aware of our research aims and responded, analyzed, and disseminated accordingly. While relational reflexivity acknowledged the collaborative nature of data formation: the main interviewer understands her prior knowledge on youth peer support, professional function as researcher and trained psychologist, and her experience of receiving care and caring for others, influenced the interview outcomes and data analysis process. Contextual-discursive reflectivity recognized the unique narratives of participants, understanding that their stories are formed by unique experiences, specific contexts, and diverse backgrounds in the field of CAMHS. Participants disclose what they want researchers to know, and we as researchers organize and retell these stories in a coherent structured manner as ‘Results’. In reality, the stories told by participants are always more unstructured than how they are represented in the results section [14].

To organize the reflective thematic analysis, we applied five steps of thematic analysis [13]. First, we familiarized ourselves with the data by actively transcribing and re-reading the field notes and transcripts. The second step entailed generating initial codes, by open coding of the interview transcripts. Two researchers, the first author (CB) and the fourth author (JG—a YPSW with research experience) coded the transcripts independently and discussed each transcript to resolve differences. During the third step we generated initial themes by identifying patterns of similarity in codes and grouping them under a central meaningful category. The fourth step entailed further enhancing, interpreting and refining the initial themes during reflective team meetings with the authors of this study. The final step involved writing the analysis and describing the data in the ‘Results’ section below.

## Results

A total of 37 participants were interviewed: 10 youth, 10 YPSWs, and 17 clinicians. Please see Table 1 for an overview of the demographic characteristics. Data saturation was achieved during the process of open coding; no additional codes were conceptualized for the last four interviews with clinicians and last three interviews with YPSWs and youth [13, 29]. Data saturation with clinicians may have taken longer due to the diversity of their clinical roles within the CAMHS context.

**Table 1:** Demographics Characteristics Youth, YPSWs & Clinicians

Variable	N = 37
*Youth*	10
Gender	
Male	1
Female	9
Age range in years (*M, SD*)	17–24 (20.6, 2.62)
*YPSWs*	10
Gender	
Male	0
Female	10
Non-binary	0
Age	
Age range in years *(M, SD)*	21–32 (24.1, 3.47)
Organization	
National Youth Council	4
ExpEx	4
Both	2
Roles YPSWs in practice	
Coaching and emotional support	8
Advocacy	8
Advisors (e.g. research)	3
Educators (e.g. teaching and training)	6
*Clinicians*	17
Gender	
Male	6
Female	11
Age range in years *(M, SD)*	21–63 (37.7, 11.48)
Organization	
LUMC Curium	12
iHUB	4
Pluryn	1
Occupation	
Psychiatrist	1
Social therapist	5
Manager of social therapists	1
Social therapist in training	2
Clinical psychologist	1
Developmental psychologist	2
Family therapist	2
Social worker/ childcare worker	2
Case Manager	1

^1^Number does not add up to 10 because a number of YPSWs took on more than one role

^2^ None of the participants identified as non-binary. Therefore it is not included in this table

Of the youth, six had previous experience of being coached by a YPSW. Youth described several mental health challenges for which they received treatment in CAMHS, including: eating disorders, dissociative disorder, giftedness, depression, addiction, posttraumatic stress disorder, suicidal ideation, self-harm and autism spectrum disorder. Moreover, all of the YPSWs included in the study had undergone at least one training related to youth peer support work. They indicated the following key themes were covered during the trainings: storytelling, active listening, establishing (personal) boundaries, recognizing personal qualities and avoiding pitfalls, effective communication, promoting recovery and empowerment, sharing lived experience safely and valuably, and providing support to others. Of the clinicians, 16 had previous experience of working with a YPSW. The clinician who did not have previous experience of working with a YPSW, did have experience collaborating with clinicians who utilized lived experience in practice.

## Findings

The findings from our thematic analysis are divided in two sections. The first section describes the themes and subthemes linked to the unique socio-relational contributions that YPSWs can make next to clinicians in CAMHS; and the second section explores the necessary requirements to integrate and safeguard the socio-relational contributions of YPSWs in practice. Overall, during the interviews youth tended to focus more on the socio-relational contributions YPSWs can make to CAMHS, while YPSWs and clinicians were commonly more centered on the prerequisites necessary to safeguard the contributions of YPSWs in CAMHS.

### ***Section 1: the unique socio-relational contributions of YPSWs in addition to mental health professionals in CAMHS***

The participants described four themes associated with the unique socio-relational contributions of YPSWs. These themes are explored below.

### Theme 1: the power of the relationship between YPSWs and youth

The unique relationships between YPSWs and youth can have profound positive impacts on the well-being and growth of youth.

#### Empowering youth to take control

Youth described that in relationships with YPSWs they were empowered to take control over their lives as they experienced time and freedom to talk about topics they experienced as important. For example, in relationships with YPSWs, youth felt free to play games, vent, ask questions related to recovery, and talk about topics of interest to them. Youth described they experienced less freedom in relationships with clinicians, as these are commonly bound by treatment goals, treatment protocols, and time.

“I feel like there is space to express my needs and talk about things I want to talk about with a YPSW. With a clinician, you already know what they are needed for, they are needed to achieve the goals that have been discussed for treatment.” (Young person 7)

This quote reflects this difference in how youth perceive interactions with YPSWs and clinicians. Suggesting that youth perceive YPSWs as providing a more open space to express their needs and discuss topics of personal importance. This can lead to youth taking personal initiatives and feeling empowered to take control. This may be attributed to the perception that YPSWs are more flexible in their roles and are not solely focused on treatment goals, unlike clinicians who are often bound by specific treatment objectives and protocols. The quote highlights the importance of the relational aspect of peer support, where YPSWs potentially offer a more holistic approach beyond clinical goals.

Youth also described that YPSWs empowered them by recognizing and outlining the strengths associated with their ‘vulnerabilities’.

“A YPSW can show that what you're struggling with can simultaneously be your strength. For instance, the ability to endure an addiction requires a certain level of perseverance.” (Young person 10).

The quote underlines the unique ability of YPSWs to relate to struggles of youth on a more personal level. YPSWs can validate struggles and serve as role models by demonstrating that adversity and struggle can be a source of strength and resilience. This may resonate more deeply with a young person than advise from non-peer clinicians who may not have personal experience of such challenges. This is also described in the quote below, were one clinician who worked with youth experiencing crisis, described that YPSWs were uniquely capable of empowering youth who had lost all hope for future.

“I got to know the YPSW who worked on our mental health unit, and she has had a very positive influence… In situations where youth had been struggling for a long time, undergoing treatment, and had somewhat lost hope, she was able to help some of them effectively by saying, ‘I've been through it too. It took a long time for me as well. But hang in there, better times will come.’ This gave some youth the strength to persevere… No matter how beautifully we, as non-peer clinicians, tell stories, people who have lived through those experiences naturally provide much more strength.” (Clinician 1).

YPSWs add value by empowering youth, particularly by recognizing their strengths, being open to talk about topics of interest to youth, and providing hope during challenging times.

#### Helping youth to feel heard, accepted and understood

Youth also expressed that they felt heard, accepted and understood by YPSWs. Due to shared experiences with YPSWs, youth described that they experienced less loneliness and received validation from YPSWs. Youth experienced relationships with YPSWs to be free from judgement or expectations.

“I experience there to be more recognition and understanding in relationships with YPSWs. They are easier to approach. They are not like clinicians who say, ‘Oh yes, I understand you.’ But in reality you think, ‘Do you really understand me?’” (Young person 6).

In addition, all groups of participants described that YPSWs can help to create a better understanding between youth and clinicians. For instance, YPSWs are able to form a link when clinicians and youth might experience barriers related to youth culture and language.

#### Being raw, real and authentic in relationships with youth

All groups described that inherent to the relationship between YPSWs and youth, is the ability for YPSWs to remain vulnerable and ‘real’ with youth. Unlike clinicians, YPSWs are of added value because they can disclose lived experience as part of their role. Additionally, clinicians felt that YPSWs are of added value because YPSWs can be ‘raw’ and real when they approach youth. Besides, clinicians believed that by virtue of their lived experience and training YPSWs were oftentimes capable of understanding and distinguishing which personal experiences to share to be of value to different youth.

“I think the art of youth peer support is for YPSWs to decide which experiences to share with youth to protect both their personal wellbeing and the wellbeing of the young person. That must be quite difficult, even for YPSWs” (Clinician 3).

Both YPSWs and youth emphasized that because of shared backgrounds and similarity in experiences, youth were more likely to accept empathic confrontation for maldaptive behavior from YPSWs. Which in turn enabled youth to take responsibility in recovery.

“If, a YPSW says: ‘come back when you're ready to work on your problems’ it resonates, because the YPSW understands you, and you start to realize: ‘Hey, maybe I should indeed tackle my problems.’… YPSWs know exactly when someone is lying and trying to deceive them.” (Young person 10).

### Theme 2: accepting and navigating mental health problems during treatment

As a result of personal experiences with mental health challenges, YPSWs are uniquely capable of understanding the social-emotional impacts mental health challenges can have on everyday life during adolescence. This section explores socio-emotional contributions YPSWs can make to help youth accept and navigate mental health challenges.

#### Accepting you need help

One young person who did not have previous experience with a YPSW described that she had a hard time accepting she needed help. She also did not understand her mental health challenges, and felt a YPSW with lived experience of similar mental health challenges could have helped her understand and accept her mental health challenges.

“I noticed that my therapist also didn't really understand my problems. It made me insecure, because if she didn’t even understand it well, then how am I supposed to believe that I have a problem? A YPSWs with similar problems would have been able to reassure me and help me understand my problems” (Young person 4).

Youth with prior exposure to a YPSW often shared that they perceived mental illness to be a flaw in character, and described that a YPSW helped them manage self-stigma and understand the severity of their mental health challenges.

“The moment a YPSWs makes it clear that your mental health problems are not a flaw in character, you become more open to treatment and you can start working on it” (Young person 10).

#### Navigating life in residential care settings

While youth experienced problems in understanding their mental health challenges and accepting help, those who underwent residential care (e.g., in crisis stabilization units, autism care units, addiction care clinics, and eating disorder clinics) also encountered difficulties in navigating life within residential care environments. They commonly mourned the fact that life was on hold while everyone else moved on. They felt YPSWs with lived experience of receiving residential care comprehend these challenges and were capable of helping youth grief and cope with this loss.

“I believe it's important to consider the impact hospitalization has on a young person. It's often underestimated... while your peers seem to continue with their lives, you enter a kind of a parallel world. I would look around and see my peers going to school and everything... and here I was on some mental health unit. It was intense and challenging for me. I think someone with lived experience could have made a big difference. It helps when someone acknowledges and helps you deal with these feelings” (Young person 2).

The interviewed participants also underlined YPSWs were able to create safer group environments by helping youth deal with behavior of others receiving residential care.

“I think the problem on mental health units is the toxic group atmosphere where people trigger each other to get sicker instead of better. I believe that someone with lived experience can have a much greater impact on that than, for example, a therapist because they (YPSWs) understand and notice this group toxicity, and might be able to talk about it.” (Young person 3).

#### Beyond the label

All groups described that mental health professionals commonly view youth through the lens of their diagnosis, neglecting the person behind the condition. They experienced that YPSWs were able to empower the person behind the condition by assisting them in finding their passions and strengths. They were also able to help youth cope with feelings of being ‘different’.

“When professionals label you, it gives me some kind of a confirmation, that you are different. You already feel different and the more labels you get, the more you feel like there is something wrong with you. Like I will never be socially accepted by others. In such cases a YPSW can tell you they have been in a similar situation, and that you aren’t that different. Labels aren't something that defines you”(Young person 10).

### Theme 3: empowering youth to move forward after experiencing mental health challenges

This theme explores the contributions YPSWs can make to help youth transition to places beyond child and adolescent psychiatry and youth care.

#### YPSWs instill hope for future in youth

All participants described that by virtue of their lived experience with mental health challenges, YPSW are capable of instilling hope for future in youth. Youth explained that seeing YPSWs and noting that they too had suffered mental health challenges, provided them with a role model. Youth also felt it was valuable when YPSWs described that while they were no longer suffering mental health challenges, as part of life they still experienced difficult days and faced struggles from time to time.

“For me, it was the recognition that was expressed by my YPSW, and seeing that no matter how far and deep you’ve gone, you can still find your way back. It doesn’t necessarily mean that growing up in youth care defines you, because even after that, you can create your own path.” (Young person 9).

#### Navigating mental health challenges in society

All participant groups also described that YPSWs are uniquely capable of linking youth back to society. Youth explained that it can be challenging to let go of the control and structure offered in residential care. Moreover, two young people described they would have benefitted from a YPSW when they were transitioning back to outpatient care and school. Especially since they struggled with disclosing about their mental illness to people at school, stigma, gossip surrounding their absence, and whether or not to cover self-harm scars in public. Clinicians also described how youth benefited from a YPSWs as they prepared to leave residential care.

“When a young person goes home or to another place in the community, they benefit from talking to a YPSW about these transitions. YPSWs possess firsthand experience in navigating such situations and can provide valuable support during these periods.” (Clinician 8).

#### Embrace stillness

While empowering youth and instilling hope in youth is an important aspect of youth peer support, youth also valued YPSWs who embraced periods of stillness during recovery. Youth felt that in relationships with clinicians they often had to work towards treatment goals, while YPSWs understood that at times they needed to stand still to reflect and pause before they could move on.

“Clinicians often want you to do well, but sometimes you need to pause and go through a tough period before things can get better” (YPSW 3).

### ***Section 2: requirements safeguard unique socio-relational contributions of YPSWs in practice***

The section Requirements safeguard unique socio-relational contributions of YPSWs in practice explores the prerequisites with regards to YPSWs and mental health organization to safeguard the socio-relational contributions of YPSWs in practice. This section is divided in two themes.

### Theme 4: prerequisites for YPSWs when working with youth with mental health challenges

During the interviews the participants described several qualities of YPSWs to work with youth in treatment settings. These qualities are described below.

#### Stability

All participants described that for YPSWs to be of added value in treatment settings, they needed to have reached stability in recovery. This was described as YPSWs having reached a point in recovery where they can both reflect, and be physically, mentally, and emotionally present for others (space holding). YPSWs and clinicians also expressed that to be of added value, YPSWs need to be able to recognize and set personal boundaries.

“I think it is important that you are not triggered by others. You should be able to reflect back on your problems. You should be fully present for the other person. For example, you should not secretly seek confirmation for your own story” (YPSW 2).

While most participants agreed YPSWs needed to be able to ‘hold space’ and set personal boundaries for others; some participants stated full absence of mental illness was not a requirement for the YPSW role in treatment settings. In fact, YPSWs and clinicians argued that recency of lived experiences with mental illness allows YPSWs to be pure and empathize more with youth. Moreover, one clinician argued that when we set standards for recovery for the YPSW role, we dehumanize YPSWs. She described suffering to be part of human life, and believed YPSWs should have access to mentors within the organization to discuss these difficult periods.

#### Age and background YPSWs

In terms of age, participants agreed YPSWs should have relatively recent lived experience with recovery in CAMHS to be of value. Both YPSWs and clinicians described YPSWs between the ages of 18 and 30 could better connect with youth because of their clear understanding in issues faced by youth in today’s society and youth culture.

“I noticed that for us it is beneficial when YPSWs are somewhat younger. I think the youth we treat identify more with someone who is between the age of 20–30, compared to someone who is 50” (Clinician 14).

The opinions regarding similarity in experiences and background between YPSWs and youth varied among the participants. Some youth preferred it when the YPSW had also struggled with addiction, depression or an eating disorder. While others described they felt a connection with YPSWs by virtue of the experiences associated with mental illness, such as isolation, feeling like a burden, facing stigma, and experiencing hopelessness. However, all participants agreed that there should be a match between the YPSW and the young person.

### Theme 5: prerequisites for organizations and healthcare professionals to safeguard the unique socio-relational contributions of YPSWs in CAMHS

This theme explores the prerequisites on organizational level to safeguard to the socio-relational contribution of YPSWs in practice.

#### Treatment climate

To successfully embed YPSWs and safeguard their contributions next to their clinicians, it is important the treatment climate is suitable for the involvement of YPSWs. For example, one clinician in child and adolescent psychiatry described that the YPSWs matched well with the existing treatment climate on the unit. A treatment climate that valued autonomy and youth centered care.

“I would like to say that I think the treatment climate, so the culture within our care unit, fits quite well with youth peer support... we value the attitude of standing by a young person and constantly considering their autonomy. We tell youngsters we are there for them, however they have to take the steps. We take a non-violent resistance approach. In my experience the YPSW we work with fits well within this climate” (Clinician 3).

#### Flexibility

Moreover, in order to be available for youth, it is important YPSWs can be flexible. For instance, scheduling sessions with YPSWs might be helpful for some youth, but many youth expressed that they preferred not to set up appointments with YPSWs. These youth preferred approaching YPSWs casually when they wanted to talk, walk or play a game.

#### Shared goals for youth peer support

Finally, all participants underlined that for YPSWs to be of added value coordination and shared goals are important. At management level, there should be protocols to ensure fair pay for YPSWs, and to clearly formulate the division of tasks between YPSWs and clinicians.

“I think it's especially important that everyone within an organization is on the same page. There should be no confusion on goals for YPSWs, because that can be uncomfortable for youth and a YPSWs… There should be a clear understanding of the goals with regard to youth peer support” (Young person 7).

## Discussion

This study investigated the unique contributions of YPSWs alongside clinicians in CAMHS, and identified prerequisites to maintain the socio-relational focus of YPSWs in practice. Key findings reveal YPSWs empower youth by promoting autonomy, valuing stillness in recovery, and recognizing strengths associated with vulnerabilities. Youth also reported reduced loneliness, increased validation, and non-judgement by YPSWs due to shared experiences. Moreover, authenticity and realness in YPSW relationships fostered trust and improved rapport between youth and YPSWs. The results indicate YPSWs assist youth by accepting their need of help, managing mental illness, and navigating life inside and outside of residential care settings and beyond classifications. The results regarding authenticity, empowerment, non-judgement, trust and validation in relationships with YPSW are in line with previous studies on youth peer support [2–8, 10, 12, 19]. Whereas the findings related to YPSWs valuing stillness in recovery and YPSWs assisting youth in navigating life in residential care settings, seem to add to the existing evidence base.

In addition to the socio-relational contributions of YPSWs, this study identified prerequisites to safeguard the integration and socio-relational contributions of YPSWs in practice. Although YPSWs did not have to have reached full ‘recovery’, they needed to attain stability in recovery to be emotionally, physically and mentally present in relationships with youth and colleagues. Moreover, clinicians and YPSWs emphasized the importance of recent lived experiences with mental health challenges for YPSWs to connect with youth in similar situations. However, our results showed a discrepancy regarding the requirements for similarity in backgrounds between YPSWs and youth. Some participants described it was a requirement to have similar mental health challenges and experiences, while others did not state this as requirement for YPSWs to form a meaningful connection with YPSWs. To our knowledge this discrepancy is less obvious in the existing literature. Most studies seem to underline peer work is based on principles of mutuality between people who share similar mental health conditions and life experiences, allowing for empowerment, role modeling and an experiential learning journey [19–21]. Therefore, our results add to the current body of literature by highlighting that shared experiences between youth and YPSWs aren't always a prerequisite. Instead, a shared understanding of the patient experience, coupled with the vulnerability, isolation, and powerlessness linked to this identity, can serve as a basis for cultivating a meaningful and trustworthy connection between youth and YPSWs. With regards to integration of YPSWs in CAMHS, it is crucial for YPSWs to possess a profound understanding of the patient experience, including the associated feelings of vulnerability, isolation, and powerlessness, when being employed. On an organizational level, prerequisites to integrate YPSWs and safeguard the socio-relational contributions of YPSWs included a treatment climate that warrants the inclusion of YPSWs (e.g. a treatment climate taking a non-resistance approach to youth), resources to enable YPSWs to be flexible to meet the diverse needs of youth, and shared goals stemming from management to guide the involvement and pursuit of youth peer support. The results regarding organizational prerequisites, specifically regarding the need for shared goals and an organizational culture valuing youth peer support, are in line with the existing evidence base on youth peer support [2, 22].

Overall, during the interviews youth tended to focus more on the socio-relational contributions YPSWs can make to CAMHS, while YPSWs and clinicians were commonly more centered on the prerequisites necessary to integrate and safeguard the contributions of YPSWs in CAMHS. While this discrepancy may be due to different questions asked to different groups of participants (e.g. focus on partnership between YPSWs and clinicians in interviews with YPSWs and clinicians); this difference in focus can also be explained by the experiences and backgrounds of the participants. The backgrounds of youth are commonly more positioned on the relationships they have with peers, clinicians and YPSWs in CAMHS; while the experiences of YPSWs and clinicians are frequently focused on providing care and adhering to organizational guidelines in CAMHS. It is therefore likely that youth, who receive support services, often prioritize the direct quality of their relationships with peers, clinicians, and YPSWs, over indirect care provision and organizational guidelines. In contrast YPSWs and clinicians, who provide care are more likely to focus on the organizational and practical concerns related to YPSW involvement, such as training, supervision, and adherence to organizational guidelines. As professionals working within CAMHS, they are responsible for ensuring the effectiveness and safety of YPSW contributions, so their emphasis on the prerequisites necessary to safeguard these contributions is understandable.

Some of the results require further discussion. First, our results point to the added value of YPSWs beyond the traditional medically oriented focus common in CAMHS. During the interviews, youth described challenges with regards the focus on ‘labels’ (psychiatric classifications), finding their way back to society after receiving residential care, and the goal-oriented focus of relationships with clinicians. Youth valued YPSWs for their ability to provide recovery-oriented care by taking a holistic approach towards youth and contextualizing psychological suffering beyond mere classification. They underlined that YPSWs are capable of recognizing their needs, improving trust, and validating; factors which Sonderman et al. [23] describe to be important in managing a poor group climate in residential care (e.g. distrust between staff and youth, and among peers). Thus, it is not surprising that youth suggest YPSWs can contribute to the establishment of recovery-oriented climates in CAMHS. The authenticity of YPSWs enhances their approachability and allows YPSWs to empower youth by acknowledging their struggles, while simultaneously normalizing these struggles and seeing youth holistically beyond mental health challenges. The capacity of YPSWs to empower and guide youth transitioning back into society post-residential care also allows for a research proposition: there is a need for research into organizational guidelines required to link available clinical youth peer support programs to community-based peer support programs to assist youth upon discharge from CAMHS. A previous study of a transitional discharge model with peer support workers for adults discharged from acute wards has yielded positive results; adults reported fewer symptoms, enhanced quality of life, and were less likely to be readmitted [28]. Further research into the requirement for transitional discharge models linking clinical YPSWs to community-based YPSWs is necessary, as our findings suggest that YPSWs likely have the ability to enhance the recovery of youth within communities after discharge from residential care.

Another result that warrants further discussion concerns the prerequisites for YPSWs to work in CAMHS. On one hand, participants described YPSWs should have attained stability in recovery to add value to youth. While on the other hand, participants expressed YPSWs should have recent experiences with mental health challenges to improve relatability with youth. The tension between having recent lived experience, while at the same time needing to have reached stability in recovery to empower youth, is also described as a dilemma in previous qualitative studies [18, 24]. These studies underline the pressure of YPSWs to be recovery ‘role models’ and ‘helpers’, while also needing to put forward their youthfulness and lived experiences [18–24]. The youthfulness and recency in lived experience are core components of the YPSW role, allowing YPSWs to add value beyond medical oriented practice models. Thus, to maintain stability in recovery and manage the tension associated with the YPSW role, we agree with previous research that YPSWs should have dedicated training and supervision, preferably from peer staff, to assist YPSWs in developing professional skills, and recognizing and setting (personal) boundaries linked to peer support work [18, 24, 25]. In line with Delman and Klodnick (2016) [22], regular ongoing clinical supervision can support YPSWs in exploring their previous adversities (e.g. trauma and stigma) alongside present job challenges. This process enables YPSWs to critically analyze these difficulties within a professional setting and consider how they can use these insights to support young individuals. As well as supervising YPSWs, clinical staff also benefit from additional training and supervision to facilitate the integration of YPSWs. This support assists clinical staff to understand their inclination to take on the role of a therapist in their interactions with often young YPSWs, potentially impacting the job performance and accountability of YPSWs [22].

## Strengths and limitations

Our study has several strengths. First, while the focus of our study was on youth peer support, the results are more extensive and indicate broader challenges faced by youth receiving treatment in CAMHS. For example, letting go of the structure and control offered by residential treatment; and challenges youth experience linked to receiving labels (classifications). Insight into these broader challenges faced by youth can inform future research and interventions for youth in CAMHS. Another strength in our study is the involvement of YPSWs during the formulation of the interview topics. Involving YPSWs during the development of interviews topics ensured that the topics cover a wide range of issues relevant to YPSWs in CAMHS [26]. A final strength of this study is that we interviewed and analyzed perspectives from three groups of participants (youth, YPSWs and clinicians) to gain insight in the differing viewpoints surrounding youth peer support. This provided us with a comprehensive view of the added value of youth peer support and the essential prerequisites from three key groups, all of which are crucial during the integration process of YPSWs in CAMHS. We recommend future research to conduct observational studies to gain a deeper understanding of the interplay between these key groups during the integration process of YPSWs in CAMHS.

It is important to consider some limitations when interpreting this study. The first limitation concerns the overrepresentation of female participants, especially among youth and YPSWs. This gender ratio is not the ratio we encounter in practice, as the representation of male and females in CAMHS is suggested to be similar [27]. Another limitation encountered in our study is the convenience sampling approach taken, which limits our results to a group of participants with potential strong opinions regarding youth peer support. A third limitation encountered in our study is the diversity in backgrounds of youth, YSPWs and clinicians; the groups of participants included received treatment or worked for different organizations. While this diversity allows for various perspectives on youth peer support, it also increases the likelihood that some contextual nuances (e.g. due to organizational, systemic, individual, and cultural differences) related to organizations employing YPSWs are lost. We urge for additional case studies to explore these contextual nuances, and gain more insights in the barriers and facilitators in relation to the pursuit of peer support programs in practice. A third limitation is related to the youths that participated in our study. Four youths did not having previous experience of working with a YPSW. Therefore, these youths might have projected their care needs and expectations for care onto their perceived added value for YPSWs. Hence, future research should account for this limitation by examining the perceptions of youth towards YPSWs prior to and after exposure to YPSWs. Moreover, the age range of the youths approached and interviewed was 12–24 years old, whereas CAMHS commonly offers care to youths up to the age of 21. As a result, the experiences of youths aged 21 and above may differ from those of the younger participants. However, we have chosen the age limit of 24 years because we expect that these young people are still capable of reflecting on the care they received a few years ago. Finally, in the interviews with youths, four out of ten had prior experience with one of the YPSWs who was also a co-researcher during this research project. This dual relationship may have led to social desirability bias, whereby the participant may have felt inclined to provide socially desirable responses to interview questions. However, there was no peer support relationship between these participants and the co-researcher; and the first author (CB) contacted these youths separately to ensure that they were comfortable with the co-researcher’s participation during the interviews.

## Conclusion

In conclusion, this study explored the unique socio-relational contributions and prerequisites for YPSWs in CAMHS. Underlining YPSWs add value beyond traditional medical oriented models by employing youthfulness, lived experience and authenticity to empower and build trusting relationships with youth. To drive forward transformation of CAMHS in the upcoming years with YPSWs, adequate guidelines, fitting treatment climates, and shared goals for youth peer support are required.

## Supplementary Information

Below is the link to the electronic supplementary material.

Supplementary file1 (DOCX 24 KB)

## Data Availability

The datasets of the current study are available from the corresponding author upon reasonable request.
