# Supplementary material for: A balancing act: integrating the expertise of youth peer workers in child and adolescent mental health services
Source: Eur Child Adolesc Psychiatry. 2024 Jun 15;34(1):327–39. doi: 10.1007/s00787-024-02498-4 (PMC11805828; doi:10.1007/s00787-024-02498-4)
Supplement: Supplementary file 1 — Supplementary file1 (DOCX 24 KB) [file 787_2024_2498_MOESM1_ESM.docx]

**Appendix A: Topic lists and sample interviews**

***Topic list youth peer support workers***

| **Topic list for youth peer support workers** | **Example questions** |
| --- | --- |
| **Introduction** | Information regarding research project and process of the interview, informed consent, and voluntary nature of the interview. |
| **Motivation to participate and background information** | - Can you tell me more about why you decided to participate in the interview today? - How old are you? - Can you tell me more about your work as a YPSW? What does a typical day entail? - Why did you choose to work as a YPSW in the in youth-serving context? - Why do you think it is important to involve YPSWs in practice? |
| **Added value of youth peer support** | - Have you ever worked with a (youth) peer support worker yourself? Yes: How did you experience this? No: would this have been helpful for you? - Are there areas in your work that are going well? Can you tell me more about this? - Do you ever get compliments from young people and/ or colleagues for the work you do? If so, what type of compliments? - How would you describe the added value of youth peer support? - What types of unique contributions can YPSWs make to CAMHS?   - (potential) additional questions to prompt discussion:     - What types of unique contributions can YPSWs make to youth in CAMHS?     - What types of unique contributions can YPSWs make in treatment teams?     - What types of unique contributions can YPSWs add to the organization as a whole? |
| **Roles and age youth peer support workers** | - What type of roles can YPSWs fulfill in the youth serving context? Do you have examples? - When and for whom is it important and valuable to work with YPSWs? |
| **Training and skills youth peer support workers** | - What kind of education or training have you followed to work as a YPSW? - Did you miss subjects/ training areas/ skills during your training as a YPSW? For example: things that might have been relevant for your work today? - What type of skills are beneficial for your work as a YPSW? - If you were asked to train or educate future YPSWs, what would you tell and/ or teach them? - Does a YPSW need to have similar lived experience to the young people they support? |
| **Recovery & Age** | - How would you describe recovery? What does recovery entail to you?   - According to your definition of recovery: When would you say you are ready to work as a YPSW? Does this also depend on the type of role you are going to fulfill in the youth serving context? - When do you feel a YPSW is prepared to take on the role of YPSW in CAMHS (supporting youth during treatment)? - Is there a certain age when you can no longer work as a YPSW in the youth-serving context? |
| **Guidance, supervision and support** | - What organizational requirements need to be in place to stimulate and facilitate the employment of YPSWs? - What type of guidance and support is important for you to work well? |
| **Communication and barriers and facilitators in partnership with colleagues** | - Have you experienced barriers in the partnership with (non-peer) colleagues? - Can you tell me more about things that are going well in partnership with (non-peer) colleagues? |
| **Facilitators and barriers during implementation and pursuit of youth peer support services** | - Would you like to tell us when you started as a YPSW? How did you experience this? - Are there aspects within CAMHS that can make it more difficult to introduce and pursue youth peer support services? - Are there things in your employment that have made it difficult for you to pursue your work? - Are there factors within organizations that can hinder your work as a YPSW? |
| **Self-Disclosure** | - What is it like for you to share your own experiences with mental health difficulties and recovery with young people? - What is it like for you to share your own experiences with mental health difficulties and recovery with (non-peer) colleagues? - What do you think of clinicians who share openly about their own past with psychological complaints and adversities? - In your opinion: how does self-disclosure and personal sharing differ between YPSWs and non-peer colleagues (refers to clinicians)? - How does a YPSW differ from a clinician within the context of treatment in CAMHS? |

***Topic list clinicians***

| **Topic list for healthcare professionals** | **Example questions** |
| --- | --- |
| **Introduction** | Information regarding research project and process of the interview, informed consent, and voluntary nature of the interview. |
| **Motivation to participate and background information** | - Can you tell me more about why you decided to participate in the interview today? - How old are you? - Can you tell me more about your work? What does a typical day entail? - Can you tell me about your experience with YPSWs? |
| **Added value of youth peer support** | - How would you describe the added value of youth peer support? Do you have examples? |
| **Roles youth peer support workers** | - What type of roles can YPSWs fulfill in the youth serving context? Do you have examples? - When and for whom is it important and valuable to work with YPSWs? |
| **Training and skills youth peer support workers** | - What type of skills are beneficial for youth peer support workers to have in the youth-serving context? - Does a YPSW need to have similar lived experience to the young people they support? |
| **Recovery and age** | - How would you describe recovery for YPSWs? What entails recovery for you?   - According to your definition of recovery: When would you say a YPSW is able to be employed? Does this also depend on the type of role a YPSW is going to fulfill in CAMHS? - Is there a certain stage or age when a YPSW is no longer able to work as a YPSW in CAMHS? (ask about: policy, research, education, treatment, and face-to-face with young people). |
| **Guidance, supervision and support** | - What organizational requirements need to be in place to stimulate and facilitate the employment of YPSWs? - What type of guidance and support is important for you to work well with a YPSW? - What type of support do you think is valuable for YPSWs? |
| **Communication and barriers and facilitators in partnership with colleagues** | - Have you experienced barriers in the partnership with YPSWs? - Can you tell me more about things that are going well in your partnership with YPSWs? |
| **Facilitators and barriers during implementation and pursuit of youth peer support services** | - Are there aspects within CAMHS that can make it more difficult to introduce and pursue youth peer support services? - Are there things in your employment/ setting that can make it difficult for you to include and work with YPSWs? - Are there factors within organizations that can hinder the pursuit and employment of YPSWS? |
| **Self-disclosure** | - Do you share your own experiences with mental health difficulties and recovery with young people? - Do you to share your own experiences with mental health difficulties and recovery with colleagues? - What do you think of non-peer colleagues who share openly about their own past with psychological complaints and adversities? - In your opinion: how does self-disclosure and personal sharing differ between YPSWs and non-peer colleagues? - How does a YPSW differ from a clinician within the context of treatment in CAMHS? |

***Topic list youth***

| **Topic list for healthcare professionals** | **Example questions** |
| --- | --- |
| **Introduction** | Information regarding research project and process of the interview, informed consent, and voluntary nature of the interview. |
| **Motivation to participate and background information** | - Can you tell me more about why you decided to participate in the interview today? - How old are you? - Can you tell me more about your experience with CAMHS? What type of treatment did you receive? - Can you tell me about your experience with YPSWs?   - *In case the young person has no prior experience with youth peer support:* Could a YPSWs be helpful to you during your (past) treatment? If yes, how could a YPSW support you?   - Top of Form |
| **Added value of youth peer support** | - *Youth with prior experience with a YPSW:* How would you describe the added value of youth peer support? Do you have examples? - *Youth without prior experience with a YPSW:* How could a YPSW be of value to you? Do you have examples? - *All youth:* Does a YPSWs need to have similar lived experience to the young people they support? |
| **Roles youth peer support workers** | - What type of roles can YPSWs fulfill in CAMHS? Do you have examples? - When and for whom is it important and valuable to work with YPSWs? |
| **Training and skills youth peer support workers** | - In your opinion: what type of skills are beneficial for youth peer support workers to have in the youth-serving context? - Does a YPSW need to have similar lived experience to the young people they support? |
| **Recovery and age** | - How would you describe recovery for YPSWs? What entails recovery for a YPSWs you?   - According to your definition of recovery: When would you say a YPSW is able to be employed as a YPSW? Does this also depend on the type of role a YPSW is going to fulfill in CAMHS? - In your opinion: Is there a certain stage or age when a YPSW is no longer able to work as a YPSW in CAMHS? (ask about: policy, research, education, treatment, and face-to-face with young people). |
| **Introduction of YPSWs, guidance, and organizational factors surrounding youth peer support** | - What organizational requirements need to be in place to stimulate and facilitate the employment of YPSWs? - What type of (organizational) support is valuable for YPSWs? - Are there things you would (have) want(ed) to know about peer support prior to working with a YPSWs? - Are there aspects within CAMHS that can make it more difficult to introduce and pursue youth peer support services? |
| **Communication and barriers and facilitators in partnership between YPSWs and youth** | - *For youth with prior experience with a YPSWs:*    - Have ever had negative experiences with a YPSWs? Can you give an example?   - Have you ever had positive experiences with a YPSWs? Can you give an example? - *For youth without prior experience with a YPSWs*:   - Are there things you would not appreciate if you were receiving support from a YPSW?   - What are the aspects a YPSW could assist you with? |
| **Self-disclosure** | - What do you think of clinicians who share openly about their own past with psychological complaints and adversities? - What do you think of YPSWs who share openly about their own past with psychological complaints and adversities? - In your opinion: how does self-disclosure and personal sharing differ between YPSWs and non-peer colleagues (refers to clinicians)? - How does a YPSW differ from a clinician within the context of treatment in CAMHS? |
